# Divergent range dynamics among non-native Coleoptera: climate drivers and poleward expansion in multiple species

**DOI:** 10.3389/finsc.2026.1917683

**Published:** 2026-09-17

**Authors:** Stella M. Januario, Carmen P. Silva, Daniela N. López, Sergio A. Estay

**Affiliations:** Facultad de Ciencias, Instituto de Ciencias Ambientales y Evolutivas, Universidad Austral de Chile, Valdivia, Chile

**Keywords:** climate change, Coleoptera, non-native insects, range dynamics, Southern South America

## Abstract

**Introduction:**

The expansion and contraction of species ranges result from population dynamics, species interactions, and environmental responses. As temperatures rise at high latitudes, some temperate species may be shifting poleward. As non-native species expand in range they threaten native biodiversity and disrupt ecosystem functioning, making range dynamics critical to understand for effective control programs.

**Methods:**

This study examined the dynamics of the southern geographic limit of non-native Coleoptera species in Chile. Using occurrence data from 25 species recorded between 2007 to 2022, we evaluated how the southern range limit of each species responds to environmental variables, and whether the observed southward displacements are consistent with the regional climate trends.

**Results:**

Model outputs provide significant evidence that the southern range limit dynamics of at least 11 species are linked to seasonal and annual fluctuations in temperature and precipitation. In addition, nine species already showed significant southward expansion, with displacements ranging from 200 to 1400 km between the minimum and maximum recorded southern limits.

**Discussion:**

The results shed light on the range dynamics of non-native species in the southern cone of South America and can help design containment strategies that minimize the impacts of these non-native species on austral, more pristine ecosystems.

## Introduction

The geographic range is the area occupied by a species at a given time (1, 2). Measured as the area of occupancy, the specific locations a species inhabits, or as the extent of occurrence (3), it is a key and dynamic characteristics of a species that change across space and time, driven by ecological and evolutionary factors (4, 5). Such changes, described as contractions, expansions, or shifts (4, 6) have been documented in many taxa, including birds, mammals, fish, plants, insects, and algae (e.g. 7, 8).

In a global context, climate change is currently a major factor influencing the geographic distribution of species (6, 9). The rise in global temperatures and changes in rainfall patterns are two direct effects pressuring organisms, communities, and ecosystems (10, 11). Several studies showed that the redistribution of organisms in response to changes in environmental conditions alters ecosystems by, among other things, altering food webs, species assemblages, species composition, and species interactions (8, 12, 13). Although many reported changes follow a predicted trend, such as poleward and upslope shifts (8, 14, 15), in other cases, species show heterogeneous responses (16–18). This makes it difficult to generalize and to predict species’ future movements, which in turn affects conservation and habitat management (19, 20).

Insects have received growing attention because many non-native species are already considered major agricultural and forestry pests worldwide (21, 22). Their economic impact is substantial, with global losses estimated at US$70 billion or more per year (23). As ectotherms, insects show diverse life-history strategies and thermal tolerances (24–26). Several traits, such as survival, growth rate, voltinism, and size at maturity, are expected to respond to changes in temperature and precipitation (27, 28). For non-native species, climate change can also modify or even eliminate biotic and abiotic constraints, creating conditions that increase their chances of expansion and/or becoming invasive (20, 29). Therefore, climate change trends are likely to exacerbate these impacts by further facilitating the establishment and spread of invasive insects. Consequently, predicting how their ranges will change is a challenge.

Although most empirical data on range shifts come from studies conducted in the Northern Hemisphere, there is growing evidence in Chile of distributional changes and new local outbreaks of native and non-native insects (22, 30). Since the 1950s, the arrival of non-native species in the region has intensified, driven mainly by globalization, agricultural expansion, and the use of biological control programs (31). In addition, central–southern Chile has faced a severe and prolonged drought for at least 14 years (32), which has reduced precipitation and water availability (33). Such conditions can weaken host plants and forests, increasing their vulnerability to pests and the expansion of some species (30). This study examines the dynamics of the southern range limits of non-native Coleoptera in Chile, their association with environmental conditions, and whether the observed southward displacements are consistent with regional climate trends.

## Methods

### Data

#### Insect data

Data on insect species richness correspond to trap records obtained from the National Plant Health System of the Agricultural and Livestock Service (SAG, in Spanish), Ministry of Agriculture of Chile, as reported by SAG (34). Sampling sites are located in several environments, including urban parks, forest plantations, and natural, semi-natural, or rural areas. According to SAG procedures, trap samples were transported to official laboratories, and all species’ incidences were recorded in the National Database (35, 36). Sampling sites and sampling effort were maintained relatively constant through time. In this study, only records of exotic species with a long history of establishment in Chile were used, to avoid species in the expansion phase after introduction (25 species, with over 100 records per species, **Figure 1**), collected between 2007 and 2022. Taxonomic classification, native range, feeding habits, host plants, and habitat preferences for each of the 25 species are provided in **Supplementary Table S3**. Also, spatial distribution maps showing occurrence records are provided in **Supplementary Figure S2**. To analyze the data in a biologically meaningful way, the dates were reclassified into growing seasons. In Chile, the growing season spans from July of one calendar year to June of the next, reflecting the Southern Hemisphere’s seasonal cycle. For example, season 2021 corresponds to records collected between July 2021 and June 2022.

**Figure 1:**
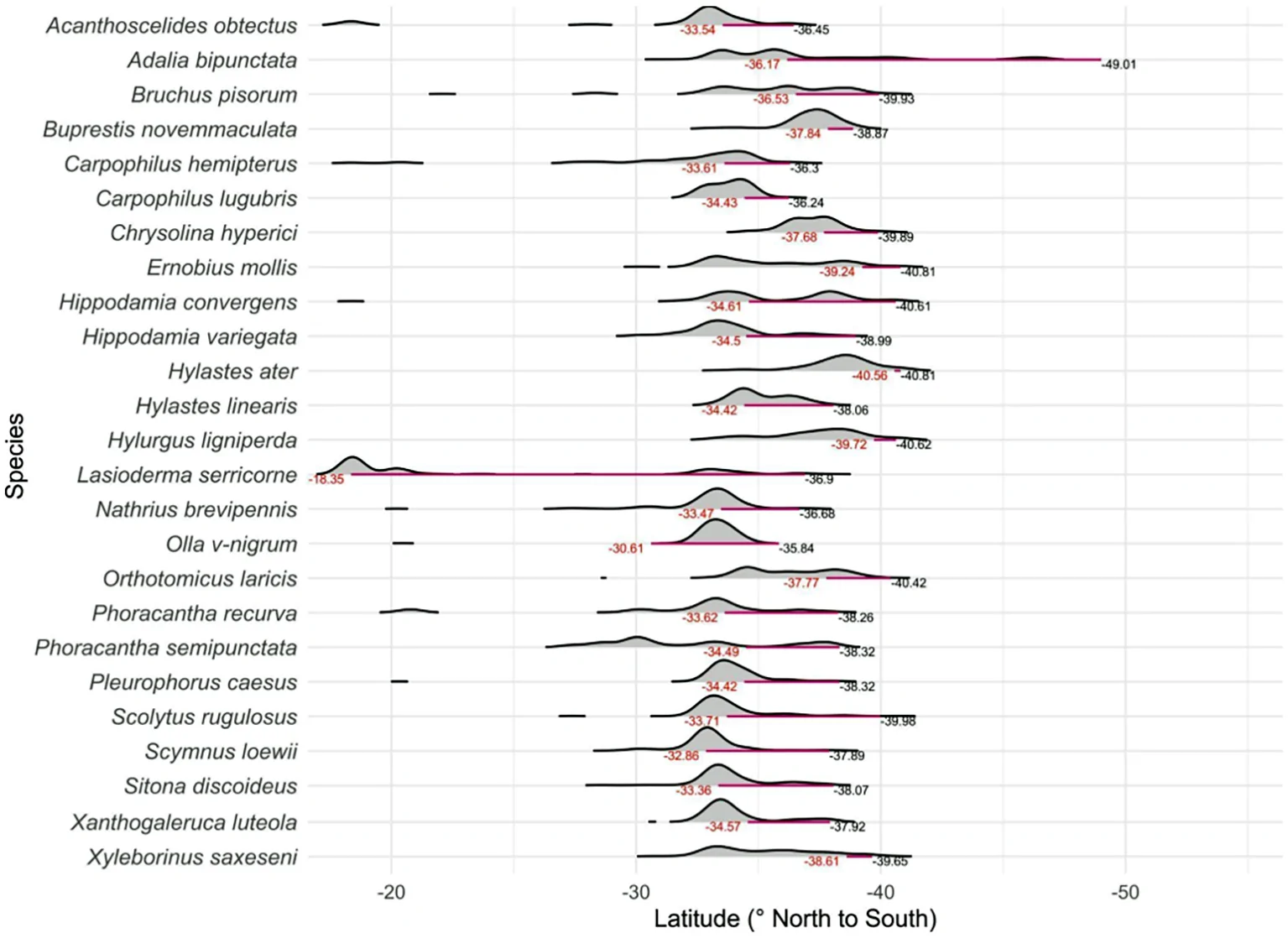
Density graphs showing the latitude variation for each Coleoptera species. The minimum southern latitude is indicated in red and the maximum in black. The range of latitude between these two points is highlighted in dark pink.

Given Chile’s width (90–445 km), the analysis was focused on the latitude of the southern limit reached by the species each season. The northern limit was not analyzed because the Atacama Desert is a major barrier that limits the dispersal of these insects. Changes in the southern limit across seasons were evaluated for their association with seasonal temperatures and precipitation, and whether the southern limit of each species has expanded consistently poleward over the time window considered in the study. All records were geolocated using EPSG 32718 projection to express coordinates in meters. Finally, for each species and season, the 97.5% quantile latitude was defined. This description of the southern limit was used to avoid noise from records without confirmed establishment (e.g., anecdotal records in ports due to cabotage). When a specific species-year combination had too few points to calculate the percentile, it was excluded from the final analysis.

#### Environmental data

Temperature and precipitation data were obtained from ERA5 (37, 38) monthly-averaged 2m temperature and total precipitation (0.25° pixel size). Using these data, isotherms and isohyets were calculated for each season. Seasons in the Southern Hemisphere were defined as follows: summer (January, February, and March), autumn (April, May, and June), winter (July, August, and September), and spring (October, November, and December). In the first step, isotherms were calculated at two temperatures (12 °C and 18 °C) in spring (HL12 and HL18, hereafter) and summer (L12 and L18, hereafter). These thresholds were selected because they correspond approximately to the median and 90th percentile of the lower thermal limits for beetles reported by Jarosik et al. (39). Precipitation data were then processed to obtain isohyets at 300 and 500 mm (PP300 and PP500, here after). These values ​​correspond to the approximate range of annual rainfall in central Chile and can serve as proxies for drought stress at the southern distribution limits of these species (40). Finally, the latitude of the southernmost point for each isotherm and isohyet was calculated from annual and seasonal data using the EPSG:32718 projection (WGS 84/UTM zone 18S), which allows distance calculation in meters. This approach enabled evaluation of the relationship between the species’ southern limit and the southernmost position of each isotherm and isohyet. The regional trend of each variable is shown in **Supplementary Figure S1**.

#### Data analysis

To model the relationship between the species’ southern limits and climatic variables, Generalized Additive Models (GAM) were used, because they facilitate the detection of nonlinear relationships (41). Several strategies were applied to avoid overfitting, a common problem in GAMs. The number of variables in each initial model was first reduced, constraining each model to only three variables: one of the two spring isotherm latitudes (HL12, HL18), one of the two summer isotherm latitudes (L12, L18), and one precipitation (PP300, PP500). Cubic regression splines with shrinkage were also used (41), which add an extra penalty to the model; if the penalty is high enough, all smoothing coefficients are shrunk to zero. In this case, the effective degrees of freedom of the variable(s) become so small that they can be removed (41). In the same vein, Restricted Maximum Likelihood (REML) was used instead of Generalized Cross-Validation (GCV, the default method) to estimate the smoothing parameter. The best model for each species was selected using the Bayesian Information Criterion weights (BICw) and the explained deviance (D^2^). Finally, the Average Shared Variance method (42) was used to determine the relative importance of each variable in the best model.

To evaluate potential expansions or contractions of the southern distribution limit, the association between each seasonal value (2007–2021) and the latitude of the southern limit for each species was assessed using Spearman’s rank correlation coefficient (ρ). Spearman’s ρ is a non-parametric measure of monotonic association that ranges from −1 to 1. Negative correlations were interpreted as indicating southward expansions and positive correlations as contractions, changes were considered significant when the 95% confidence interval of ρ did not include zero (43).

All analyses were performed in the Qgis (3.44.3) and R environment (44) with the packages “terra” (45), “mgcv” (41), “gam.hp” (42), and “correlation” (46).

## Results

For 11 of the 25 species: *Carpophilus lugubris, Nathrius brevipennis, Scymnus loewii, Orthotomicus laricis, Xyleborinus saxeseni, Phoracantha semipunctata, Buprestis novemmaculata, Lasioderma serricorne, Bruchus pisorum, Hylurgus ligniperda*, and *Hylastes linearis*, the best models showed deviance explained (D^2^) greater than 0.5 (**Figure 1**, **Table 1**, species with a D^2^ < 0.5 were included in the **Supplementary material Table S1**). In the best models, PP500 was the most important environmental variable for five of the eleven species (**Table 2**), followed by HL12, PP300, then HL18 and L18, and finally L12 in descending order of importance (**Table 2**, species with a D^2^ < 0.5 were included in **Supplementary Table S2**).

**Table 1:** Generalized additive models (GAMs) assessing the effect of climatic variables on the southern distribution limits of species (*S_lim*).

Species	Best model	D^2^	BIC	BICw
*Carpophilus lugubris*	*S_lim ~ 6154.3 + s(HL12, edf=2.21) + s(L18, edf=0.93) + s(PP500, edf=3.51)*	0.985	119.941	0.968
*Nathrius brevipennis*	*S_lim ~ 6185.9 + s(HL18, edf=2.85) + s(L18, edf=3.16) + s(PP300, edf=2.13)*	0.968	160.130	1.000
*Scymnus loewii*	*S_lim ~ 6165.3 + s(L12, edf=2.92) + s(PP500, edf=1.58)*	0.845	172.265	0.968
*Orthotomicus laricis*	*S_lim ~ 5668.6 + s(HL12, edf=2.03) + s(L12, edf=1.15) + s(PP300, edf=1.83)*	0.797	166.466	0.806
*Xyleborinus saxeseni*	*S_lim ~ 5657.6 + s(HL12, edf=1.61) + s(L18, edf=0.83) + s(PP300, edf=1.77)*	0.777	146.806	0.576
*Phoracantha semipunctata*	*S_lim ~ 5882.1 + s(L12, edf=1.64) + s(PP500, edf=1.76)*	0.755	177.930	0.379
*Buprestis novemmaculata*	*S_lim ~ 5737.2 + s(HL18, edf=1.81) + s(L18, edf=1.33)*	0.717	144.490	0.475
*Lasioderma serricorne*	*S_lim ~ 6573.6 + s(HL12, edf=1.58) + s(L12, edf=1.67)*	0.684	236.889	0.341
*Bruchus pisorum*	*S_lim ~ 5721.9 +s(HL18, edf=1.10) + s(L18, edf=0.84) + s(PP500, edf=1.19)*	0.649	180.746	0.573
*Hylurgus ligniperda*	*S_lim ~ 5539.7 + s(HL18, edf=1.89) + s(PP300, edf=1.55)*	0.629	151.791	0.310
*Hylastes linearis*	*S_lim ~ 6011 + s(HL12, edf=1.00) + s(PP500, edf=1.05)*	0.526	189.826	0.290

Spring temperature 12 °C isotherm (HL12), spring temperature 18 °C isotherm (HL18), summer temperature 12 °C isotherm (L12), summer temperature 18 °C isotherm (L18), precipitation 300 mm (PP300) and 500 mm isohyets (PP500). Intercept is expressed in Km. Estimated degrees of freedom for each smooth term (*edf*), deviance explained (D^2^), Bayesian Information Criterion (BIC), and Bayesian Information Criterion weights (BICw). Only species with D^2^ > 0.5 were included; the remaining species are reported in the **Supplementary Material** (**Supplementary Table S1**).

**Table 2:** Average Shared Variance (ASV) method to identify the most important environmental variables (isotherms and isohyets, see methods) affecting species’ southern limits.

Species	HL12	HL18	L12	L18	PP300	PP500	D^2^	BICw
*Carpophilus lugubris*	7	0	0	4	0	89	0.985	0.968
*Nathrius brevipennis*	0	7	0	25	68	0	0.968	1.000
*Scymnus loewii*	0	0	26	0	0	74	0.845	0.968
*Orthotomicus laricis*	32	0	20	0	48	0	0.797	0.806
*Xyleborinus saxeseni*	65	0	0	14	21	0	0.777	0.576
*Phoracantha semipunctata*	0	0	11	0	0	89	0.755	0.379
*Buprestis novemmaculata*	0	15	0	85	0	0	0.717	0.475
*Lasioderma serricorne*	55	0	45*	0	0	0	0.684	0.341
*Bruchus pisorum*	0	31	0	14	0	55	0.649	0.573
*Hylurgus ligniperda*	0	79	0	0	21	0	0.629	0.310
*Hylastes linearis*	48*	0	0	0	0	52	0.526	0.290
*N° of times most important*	2	1	0	1	2	5		

Deviance explained (D^2^), Bayesian Information Criterion weights (BICw) are shown. Only species with D^2^ > 0.5 were included; the remaining species were reported in the **Supplementary Material** (**Supplementary Table S2**). Grey cells show the most important variable for each model. *Denotes the second most important variable when the difference with the first is small.

Our results showed that nine species significantly expanded their geographical range southward; in ascending order of ρ, these correspond to *C. hyperici, C. lugubris, S. loewii, N. brevipennis, H. variegata, A. bipunctata, P. recurva, P. caesus*, and *H. linearis* (**Figure 2**). The other 16 species showed no significant change in their geographical distribution toward the south; among them, 10 showed a tendency to expand, and six to contract (**Figure 2**). The difference between the observed minimum and maximum annual southern limit varied from 1426 km for *A. bipunctata* to 202 km for *C. lugubris* (**Figure 2**).

**Figure 2:**
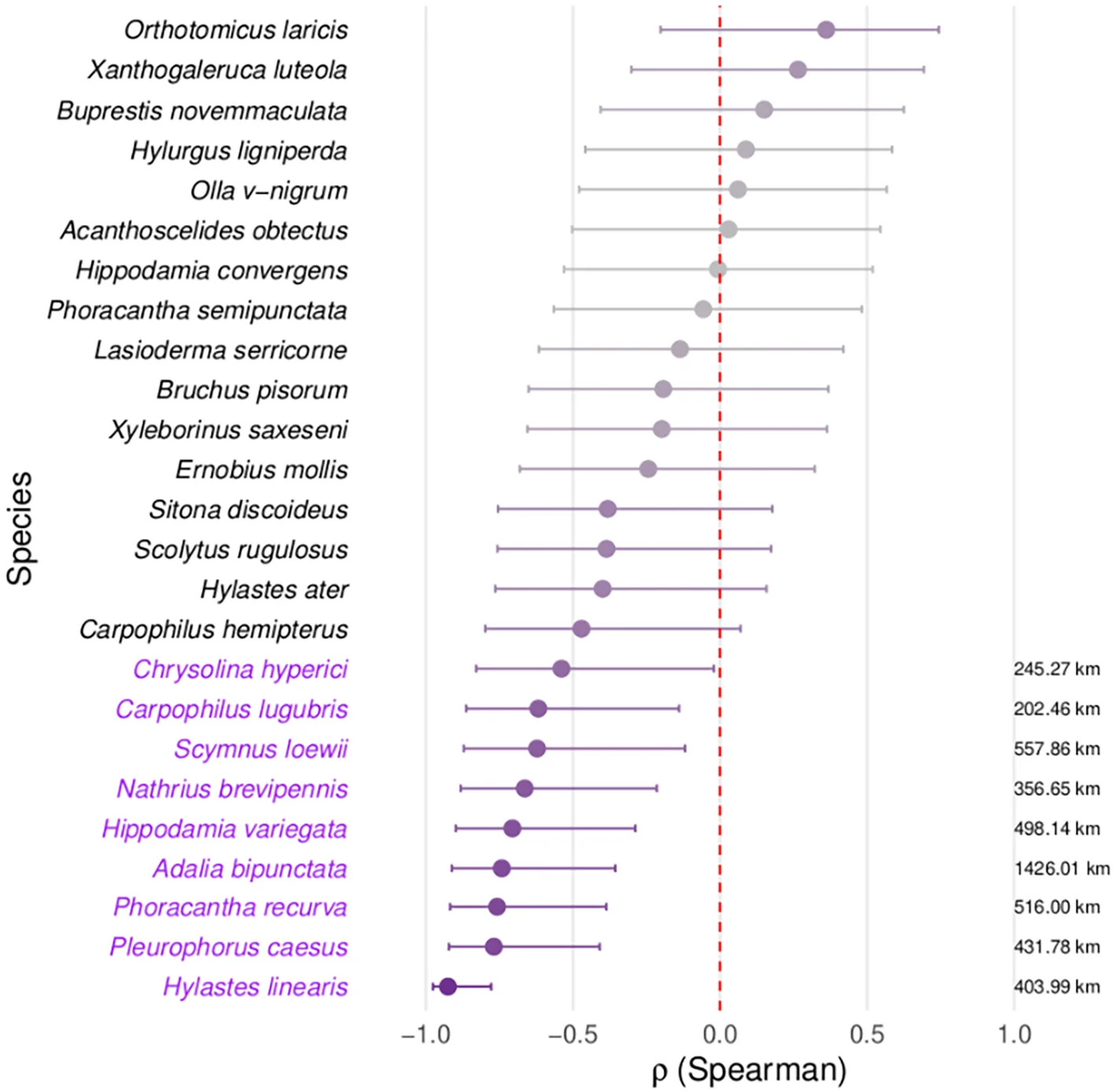
Spearman’s correlation between seasonal values (from 2007 to 2021) and the latitude of the southern limit of each species. Expansions or contractions were considered significant if the 95% confidence interval (horizontal lines) of the correlation did not contain zero (red vertical dashed line). Species in purple indicate those with significant correlations. Kilometers indicate total latitudinal shift in kilometers over the study period.

## Discussion

Just as in the case of other ectotherms which react to changes in temperature and water availability, variations in these environmental factors affect a certain number of species (27, 47). Our study did not identify a single environmental factor that accounted for the variation in the southern limit of all the species. Annual precipitation and spring temperature were the factors that best explained the southern distribution limit of 11 species of exotic Coleoptera in Chile. These species come from different families (for example, Buprestidae, Nitidulidae, Curculionidae, Cerambycidae), so generalizations based on their taxonomy cannot be made. Moreover, the species that reacted to environmental conditions include xylophagous and phloeophagous organisms, seed eaters, and predators. This suggests that the feeding guilds by themselves cannot predict how species respond to environmental changes at the southern limit. Apart from the environmental factors already analized, we also examined whether the biological characteristics listed in **Supplementary Table S3** (native range, feeding guild, host plants/habitat, and establishment time) could account for the fact that nine species expanded southward while sixteen did not. As in the case of the environmental variables, no consistent pattern emerged. The native range did not help to distinguish between the two groups: even though five of the nine expanding their range are Palaearctic (*Nathrius brevipennis, Hippodamia variegata, Adalia bipunctata, Pleurophorus caesus, Hylastes linearis*), this origin is also common among the non-expanding species (for example, *Orthotomicus laricis, Xyleborinus saxeseni, Hylurgus ligniperda*). The feeding guild was also unhelpful, even when considering species within the same family: among the aphidophagous Coccinellidae, *H. variegata, A. bipunctata*, and *Scymnus loewii* expanded, whereas the closely related *Hippodamia convergens* and *Olla v-nigrum* did not. There was also no clear connection with host association, since both narrow specialists (*Chrysolina hyperici* on *Hypericum* spp.) and generalists (*S. loewii* on aphid colonies) were found among the expanding species.

The time at which the species were established also showed a wide variation (20 to 134 years, median 44; **Supplementary Table S3**) and this did not correlate with changes in their ranges: *Carpophilus lugubris* (established in 1963) expanded its range while the closely related *C. hemipterus* (established in 1855) did not, and *N. brevipennis* (established in 1916) expanded its range while *Bruchus pisorum* (established in 1894) did not even though their establishment periods were similar.

In another important point, in the case of large poleward range expansions, human-assisted dispersal cannot be excluded, especially for more recently established species such as *H. linearis* (2006), since the spread after introduction may not be completely separated from climate-related changes. Because there is a lack of standardized data among the 25 species, other characteristics that might be relevant to range dynamics (dispersal capacity, voltinism, thermal tolerance) could not be evaluated, this being a priority for future research.

Precipitation (considering both PP300 and PP500) was the most important environmental variable explaining the change in the southern limit of seven of the eleven species with D^2^ > 0.5 (and 12 species in the complete dataset). Hawkins et al. (48) suggested that water-energy and hydric variables can explain insect richness, as these variables influence primary productivity and trophic resources. It suggests that the region’s high water availability promotes primary productivity, which, in turn, provides a large quantity of trophic resources for Coleoptera. Cepeda-Pizarro (49) also observed fluctuations in the diversity of epigean tenebrionids as total annual precipitation increased in northern Chile, suggesting that soil humidity may favor species that require humid habitats to complete their life cycles. A similar pattern of distributional variation due to changes in precipitation and temperature was observed in *Osphya*, a common genus from the Northern Hemisphere. Specifically, precipitation was mainly associated with its effects on soil conditions, which could influence the species’ life cycle (50).

The different temperature levels (L12, L18, HL12, and HL18) were the most important variables in explaining the dynamics of the southern boundary of four of the eleven species with D^2^ > 0.5 (and nine species in the complete dataset. Four species showed a better model containing only the intercept). Again, no common characteristics were observed at either the taxonomic or trophic level in this set of species. As with precipitation, temperature also imposes physiological constraints on insects. Experimental evidence of the relationship between temperature and beetle movement demonstrates how temperature affects movement from small to large spatial scales as well as from individual fitness to population survival across communities (51). For moths, warmer summer temperatures, specifically average nighttime temperatures, increase their mobility by allowing them to reach their flight threshold temperature, thereby expanding their altitudinal distribution range (52). A similar pattern was observed in peak flight for *H. ligniperda* and *H. ater* in Chile (53).

Indeed, the emergence of *Sirex noctilio* begins earlier in the season in warmer climates than in temperate climates, occurring during late December or January in South America (54). For species inhabiting temperate regions, spring temperature may represent a threshold for activity that could favor their expansion. In this context, Cortés-Contreras et al. (55) observed lower epigean coleopteran abundance in Chile during summer than during spring. Conversely, Grez et al. (56) reported greater species abundance and richness in summer than in spring in epigeic beetles.

For species that show no response to temperature and precipitation dynamics, biotic interactions may be the limiting factor constraining the southern limit. In this study case, competition and predation may show a gradient of increasing intensity toward higher latitudes, and the southern limit of these species may consequently reflect the point where biotic interactions define the boundary of the realized niche (57). Another case of biotic interactions defining latitudinal limits occurs when host availability shows a marked latitudinal gradient. In this case, the lack of food or refuge would be a better predictor of the species’ southern limit (58, 59).

Currently, the mechanisms underlying the expansion of insect pests’ geographic ranges are not fully understood (27). Among the 25 species analyzed in this study, nine significantly expanded their geographic ranges towards the South Pole. Potential mechanisms that could explain the expansion of some species include decreased winter mortality (27) and increased flight activity. For example, *H. ater* flight activity occurs at different times depending on the species’ range (53), facilitating its expansion across the country. Although we used the 97.5% quantile of latitude to minimize the effect of anecdotal detections, the possibility remains that initial long-distance dispersal followed by establishment explains the large jumps in the southern limit of some species.

Our study shows how the southern limit of some insect species can be explained using macroclimatic variables. However, for many species, macroclimatic data may be insufficient because their distribution could depend on site-specific conditions such as particular microhabitats or the presence of refugia. In such cases, more detailed environmental data will be needed to explain the range limits of these species. Integrating macro- and micro-environmental data would allow more precise predictions of range limit dynamics.

The relationship between climate change and the shift in species ranges towards the poles or higher elevations is well established (60, 61), as is the effect of warmer winters (62). If the associations between range limits and climate variables documented here reflect a genuine climatic constraint, then projected decreases in precipitation and rising temperatures in central Chile and northern Patagonia (63) suggest that southward displacement may continue — with potentially significant consequences for austral ecosystems. The establishment of native or exotic insects into these new habitats could promote changes in the local assemblages ranging from alterations in total biomass or richness to climate-induced hybridization (64). Unfortunately, few studies have explored the range limit changes in native or exotic insects in the southern cone of South America. However, because of climate change, native insects such as Ormiscodes *amphimone* have shown larger and more frequent outbreaks (65–67). Studies such as the present one aim to enhance our understanding of the factors that trigger or facilitate changes in species distribution ranges, particularly, highlighting the significant southward displacement of certain Coleoptera species in Chile.

## Data Availability

The original contributions presented in the study are included in the article/**Supplementary Material**. Further inquiries can be directed to the corresponding author.
