# Supplementary material for: Divergent range dynamics among non-native Coleoptera: climate drivers and poleward expansion in multiple species
Source: Front Insect Sci. 2026 Sep 17;6:1917683. doi: 10.3389/finsc.2026.1917683 (PMC13627314; doi:10.3389/finsc.2026.1917683)
Supplement: Supplementary file 1 [file DataSheet1.pdf]

Supplemetary material: Divergent range dynamics among non-native Coleoptera: climate drivers and poleward expansion in multiple species

Table S1: Generalized additive models (GAMs) assessing the effect of climatic variables on the southern distribution limits of species (*S_lim*). Spring temperature 12°C (HL12), spring temperature at 18°C (HL18), summer temperature at 12°C (L12), summer temperature at 18°C (L18), precipitation of 300 mm (PP300) and 500 mm (PP500). Estimated degrees of freedom for each smooth term (*edf*). Deviance explained (D^2), Bayesian Information Criterion (BIC), Bayesian Information Criterion weights (BICw). Only for species with $D^2 < 0.5$.

Species	Best Model	D^2	BIC	BICw
Hippodamia convergens	*S_lim ~ 5728.2 + s(HL12, edf=0.96) + s(PP300, edf=1.47)*	0.469	204.255	0.187
Hippodamia variegata	*S_lim ~ 6026.9 + s(HL12, edf=1.11) + s(PP500, edf=0.66)*	0.468	200.613	0.248
Ernobius mollis	*S_lim ~ 5580.4 + s(L18, edf=0.85) + s(PP500, edf=0.92)*	0.397	170.160	0.247
Phoracantha recurva	*S_lim ~ 6059.2 + s(HL12, edf=0.93) + s(PP500, edf=0.77)*	0.379	197.012	0.182
Adalia bipunctata	*S_lim ~ 5365.1 + s(HL12, edf=0.95)*	0.284	229.097	0.204
Olla_v-nigrum	*S_lim ~ 6242.9 + s(L12, edf=1.05)*	0.270	190.529	0.169
Pleurophorus_caesus	*S_lim ~ 6045.3 + s(PP500, edf=0.87)*	0.246	193.645	0.199
Xanthogaleruca luteola	*S_lim ~ 5924.1 + s(PP500, edf=0.93)*	0.243	188.512	0.152
Acanthoscelides obtectus	*S_lim ~ 6139.4 + s(PP500, edf=0.84)*	0.222	187.350	0.186
Scolytus rugulosus	*S_lim ~ 5966.7 + s(PP500, edf=0.53)*	0.103	210.965	0.139
Hylastes ater	*S_lim ~ 5496.2*	0.000	121.016	0.187
Carpophilus hemipterus	*S_lim ~ 6147.4*	0.000	184.683	0.173
Chrysolina hyperici	*S_lim ~ 5721.2*	0.000	178.362	0.171
Sitona discoideus	*S_lim ~ 6028.5*	0.000	200.675	0.125

Table S2: Average Shared Variance (ASV) method to identify most important environmental variables affecting species' southern limits such as spring temperature 12°C (HL12), spring temperature at 18°C (HL18), summer temperature at 12°C (L12), summer temperature at 18°C (L18), precipitation of 300 mm (PP300) and 500 mm (PP500). Deviance explained (D^2), Bayesian Information Criterion weights (BICw). Only for species with $D^2 < 0.5$.

Species	HL12	HL18	L12	L18	PP300	PP500	D^2	BICw
Hippodamia convergens	26	0	0	0	74	0	0.469	0.187
Hippodamia variegata	75	0	7	0	0	18	0.468	0.248
Ernobius mollis	0	0	0	51	0	49	0.397	0.247
Phoracantha recurva	58	0	2	0	0	40	0.379	0.182
Adalia bipunctata	98	0	2	0	0	0	0.284	0.204
Olla_v-nigrum	0	0	100	0	0	0	0.270	0.169
Pleurophorus_caesus	0	0	0	0	0	100	0.246	0.199
Xanthogaleruca luteola	0	0	0	0	0	100	0.243	0.152
Acanthoscelides obtectus	0	0	0	0	0	100	0.222	0.186
Scolytus rugulosus	0	0	0	0	0	100	0.103	0.139
Sitona discoideus	0	0	0	0	0	0	0.000	0.125
Chrysolina hyperici	0	0	0	0	0	0	0.000	0.171
Carpophilus hemipterus	0	0	0	0	0	0	0.000	0.173
Hylastes ater	0	0	0	0	0	0	0.000	0.187

Table S3: Summary of biological information for the species analyzed in the study. The columns includee taxonomic classification, native distribution range, year of first record in Chile, feeding habits, and host plants.

No.	Species	Family	Native range (Biogeographical Realm)	Year of first record in Chile according to López et al. (2023) and (2025)	Feeding guild	Host plants / habitat	References
22	*Buprestis novemmaculata*	Buprestidae	Palaeartic / Afrotropical	1940	Xylophagous	Pinus spp. and other conifers	FAO (2008), López et al. 2023)
4	*Phoracantha recurva*	Cerambycidae	Australasian	1970	Xylophagous (subcortical, stressed trees)	Eucalyptus spp.	FAO (2009), López et al. 2023)
17	*Nathrius brevipennis*	Cerambycidae	Palaeartic	1916	Xylophagous (dry, thin woody material)	Dry twigs and branches of various woody plants	Sláma & Berger (2006) / López et al. (2023)
21	*Phoracantha semipunctata*	Cerambycidae	Australasian	1997	Xylophagous (subcortical, stressed trees)	Eucalyptus spp.	FAO (2008), López et al. 2023)
8	*Xanthogaleruca luteola*	Chrysomelidae	Palaeartic	1994	Folivorous (adults and larvae)	Ulmus spp.	Estay (2016) / López et al. (2023)
9	*Acanthoscelides obtectus*	Chrysomelidae	Neotropical	1903	Seed feeder (also in storage)	Seeds of Phaseolus vulgaris and other Fabaceae	Artigas (1994) / López et al. (2023)
13	*Chrysolina hyperici*	Chrysomelidae	Australasian	1952	Folivorous	Hypericum spp., mainly H. perforatum	Zuñiga (1985) / López et al. (2023)
24	*Bruchus pisorum*	Chrysomelidae	Palaeartic / Afrotropical	1894	Seed feeder	Seeds of Pisum sativum	Artigas (1994) / López et al. (2023)
1	*Hippodamia convergens*	Coccinellidae	Nearctic	1961	Predator (aphidophagous)	Aphid colonies on herbaceous vegetation and crops	Zuñiga (1985) / López et al. (2023)
2	*Hippodamia variegata*	Coccinellidae	Palaeartic	1975	Predator (aphidophagous)	Aphid colonies on herbaceous vegetation and crops	Zuñiga (1985) / López et al. (2023)
5	*Adalia bipunctata*	Coccinellidae	Palaeartic	1850	Predator (aphidophagous)	Aphid colonies on trees and shrubs	Zuñiga (1985) / López et al. (2023)
6	*Olla v-nigrum*	Coccinellidae	Nearctic	2000	Predator (aphids and psyllids)	Aphid and psyllid colonies on trees	Gordon (1985) / López et al. (2013)
18	*Scymnus loewii*	Coccinellidae	Nearctic / Neotropical	1989	Predator (aphidophagous)	Aphid colonies	Gordon (2001) / López et al. (2013)
10	*Scolytus rugulosus*	Curculionidae	Palaeartic	1885	Phloeophagous (secondary; dead or stressed trees)	Rosaceae fruit trees, incl. Prunus spp. and Malus	Kirkendall (2018)
11	*Hylastes ater*	Curculionidae	Palaeartic	1980	Phloeophagous (roots and stumps)	Roots of weakened or recently dead Pinaceae; also dead trunks and large branches	Kirkendall (2018)
14	*Sitona discoideus*	Curculionidae	Palaeartic / Afrotropical	1983	Folivorous (adult); root-nodule feeder (larva)	Medicago spp. and other forage Fabaceae	Elgueta (1986) / López et al. (2023)
19	*Orthotomicus laricis*	Curculionidae	Palaeartic	1984	Phloeophagous	Pinus spp.; in Chile, plantations of Pinus radiata	Kirkendall (2018)
20	*Xyleborinus saxesenii*	Curculionidae	Palaeartic	1953	Xylomycetophagous (ambrosia beetle)	Highly polyphagous; breeds in many families of gymnosperms and angiosperms	Kirkendall (2018)
25	*Hylurgus ligniperda*	Curculionidae	Palaeartic	1981	Phloeophagous (logging residues, stumps, roots)	Pinus spp.; stumps, roots and logs, also roots of seedlings and saplings	Kirkendall (2018)
26	*Hylastes linearis*	Curculionidae	Palaeartic	2006	Phloeophagous (dead roots and stumps)	Dead roots and stumps of various Pinus spp.	Kirkendall (2018)
12	*Carpophilus hemipterus*	Nitidulidae	Afrotropical / Indomalayan	1855	Saprophagous / frugivorous	Dried and fermenting fruit; stored products	Artigas (1994) / López et al. (2023)
15	*Carpophilus lugubris*	Nitidulidae	Nearctic	1963	Saprophagous / frugivorous	Zea mays; damaged and fermenting fruit	Artigas (1994) / López et al. (2023)
3	*Ernobius mollis*	Ptinidae	Palaeartic	1963	Xylophagous (inner bark of dry conifer wood)	Seasoned coniferous wood, mainly Pinus spp.	Artigas (1994) / López et al. (2023)
23	*Lasioderma serricorne*	Ptinidae	Afrotropical / Palaeartic	1983	Stored-product feeder	Cured tobacco, dried plant products, spices	Artigas (1994) / López et al. (2023)
7	*Pleurophorus caesus*	Scarabaeidae	Palaeartic	1864	Saprophagous / detritivorous	Soil rich in decaying organic matter	Krell (2011) / López et al. (2023)

References

Artigas, Jorge N. 1994. Entomología Económica "Insectos de interés agrícola, forestal, medico y veterinario "(Nativos, introducidos y susceptibles de ser introducidos), Vol. 2.

Elgueta, M. (1986). Presencia en Chile de Sitona discoideus Gyllenhal (Coleoptera: Curculionidae). Revista Chilena de Entomologia, 14: 105-106.

Estay, S. A. (2016). Invasive insects in the Mediterranean forests of Chile. In Insects and Diseases of Mediterranean Forest Systems (pp. 379-396). Cham: Springer International Publishing.

FAO (2009). Global Review of Forest Pests and Diseases. FAO Forestry Paper, 235 p.

FAO (2008) Overview of forest pests, Chile. Forest Health & Biosecurity working papers (FBS/12E). Forest Resources & Development Service, Rome

Gordon, RD. (1985). "The Coccinellidae (Coleoptera) of America North of Mexico". Journal of the New York Entomological Society, vol. 93, no. 1, 1–912.

Gordon, R. D. (2001) South American Coccinellidae (Coleoptera). Part VII: A systematic revision of south American Scymnus (Pullus) Mulsant (Scymninae: Scymnini). Frustula Entomologica, 23 (34), 56-108.

Krell, F. T. (2011). Pleurophorus caesus New to New Mexico (Coleoptera: Scarabaeidae: Aphodiinae). Entomological News, 122(5), 491-492

López DN, Fuentes-Contreras E, Ruiz C, Ide S, Estay SA (2023) A bug's tale: revealing the history, biogeography and ecological patterns of 500 years of insect invasions. NeoBiota 81: 183-197. https://doi.org/10.3897/neobiota.81.87362

López et al. (2025) https://gbif-chile.mma.gob.cl/ipt/resource?r=exotic_insecta_chile

Sláma M., Berger P., 2006: Contribution to the knowledge of the genus Nathrius Brethes, 1916, with the description of N. cypericus n. sp. from Cyprus (Coleoptera: Cerambycidae). 11 pp. [M36] Biocosme Mésogéen 23(2)): 55-65.

Zúñiga, S. E. (1985). Eighty years of biological control in Chile. Historical review and evaluation of the projects undertaken (1903-1983). Agricultura Tecnica, 45: 175-184.

Figure S1: Temporal trends of the isotherms and isoyets through the study period. A) and B) correspond to the latitudinal position of summer and spring isotherms through time, respectively. Red line corresponds to the 18°C isotherm and the black line to the 12°C isotherm. C) Latitudinal position of annual 300 and 500 mm precipitation isohyets through time. Red line corresponds to the 500 mm and the black line to the 300 mm isohyet.

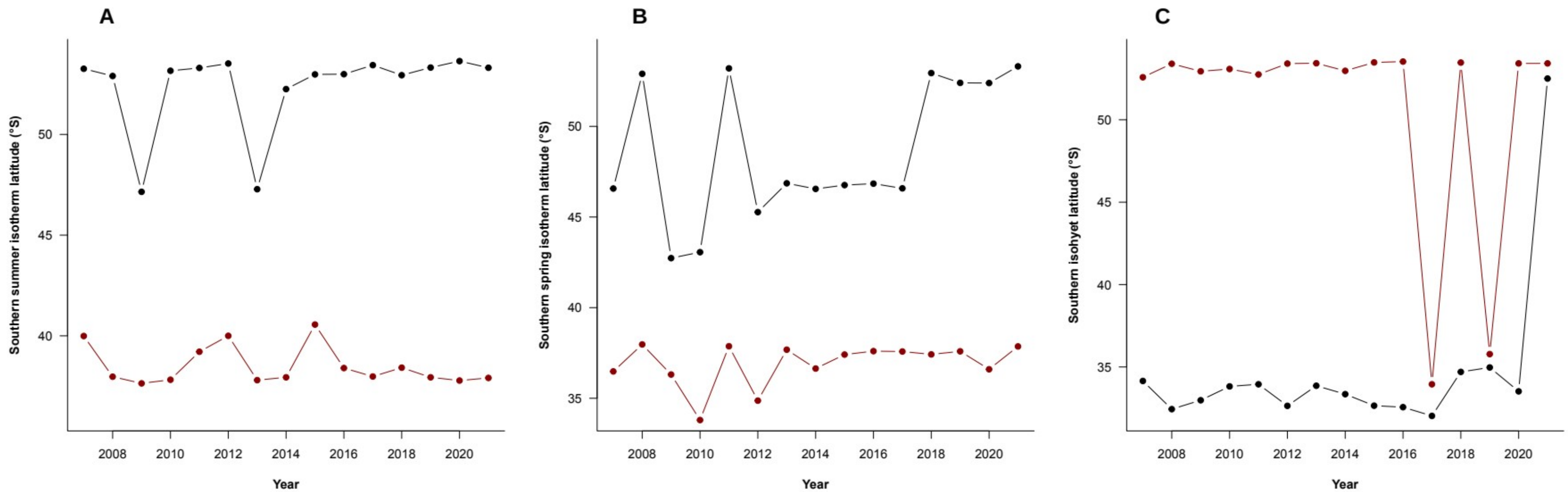

Figure S2: Maps showing occurrence records in mainland Chile for the 25 species analyzed. Red dots represent sites where the species was detected.

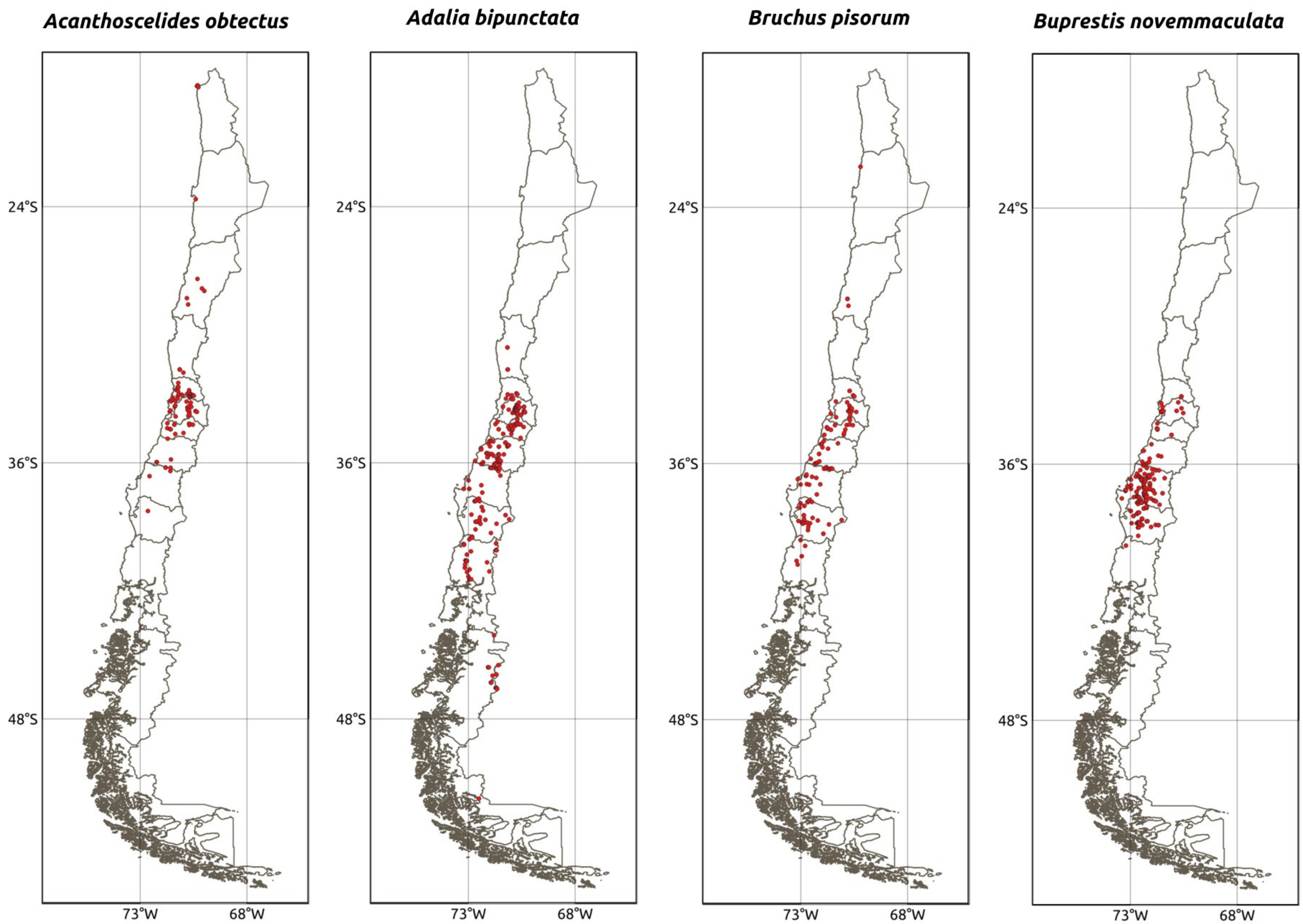
Acanthoscelides obtectus
Adalia bipunctata
Bruchus pisorum
Buprestis novemmaculata
24°S
36°S
48°S
73°W
68°W

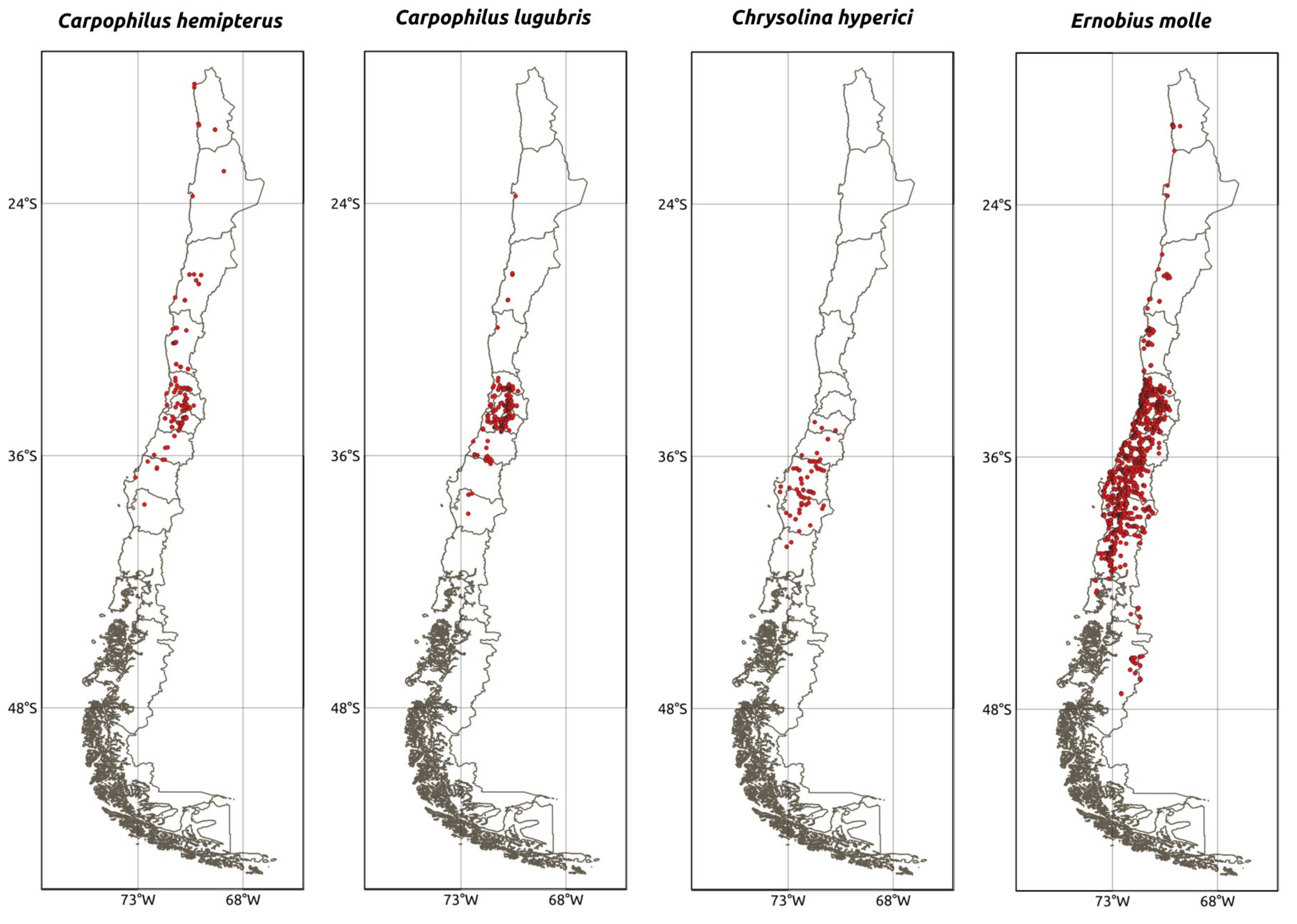
Carpophilus hemipterus
Carpophilus lugubris
Chrysolina hyperici
Ernobius molle
24°S
36°S
48°S
73°W
68°W

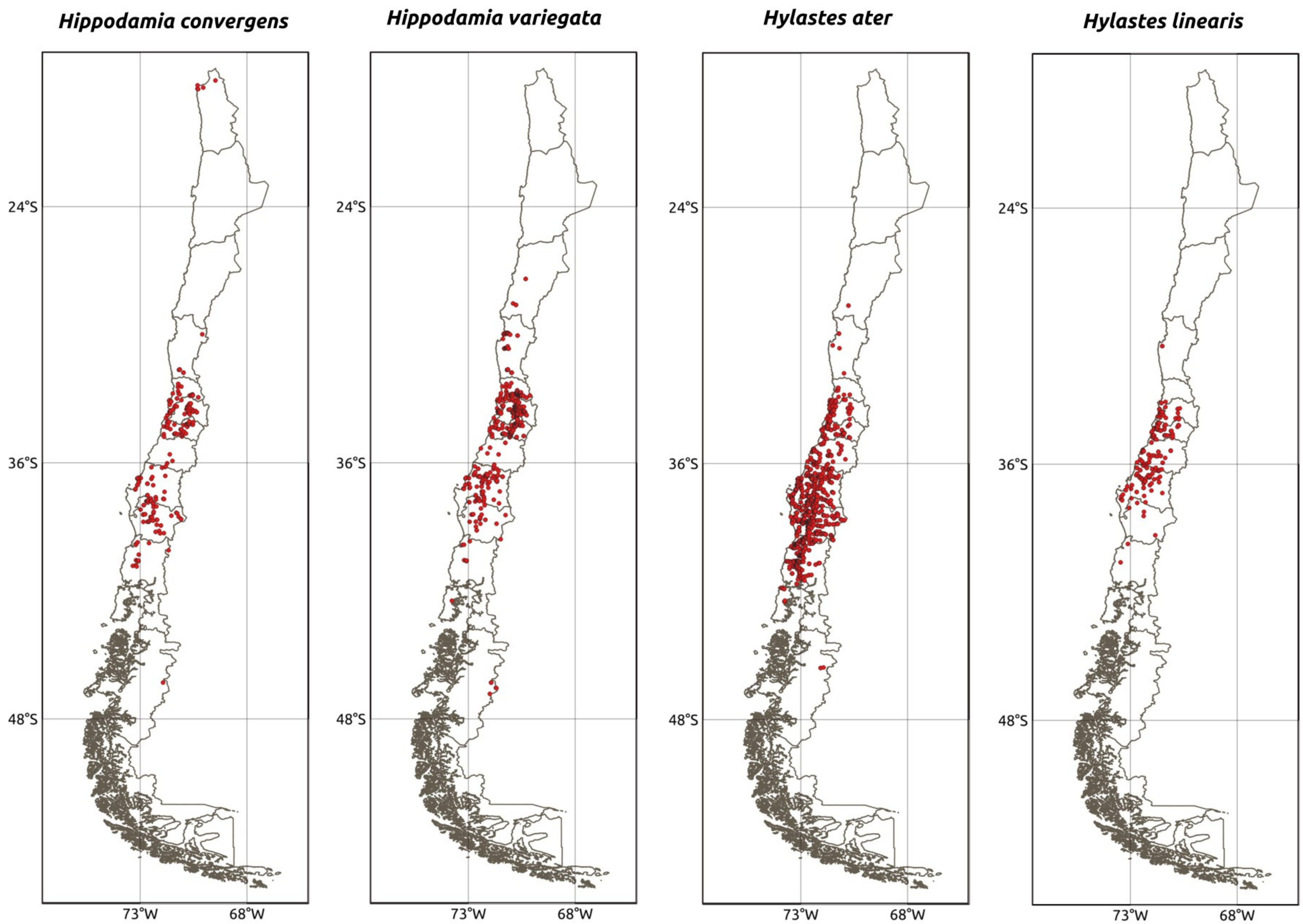
Hippodamia convergens
Hippodamia variegata
Hylastes ater
Hylastes linearis
24°S
36°S
48°S
73°W
68°W

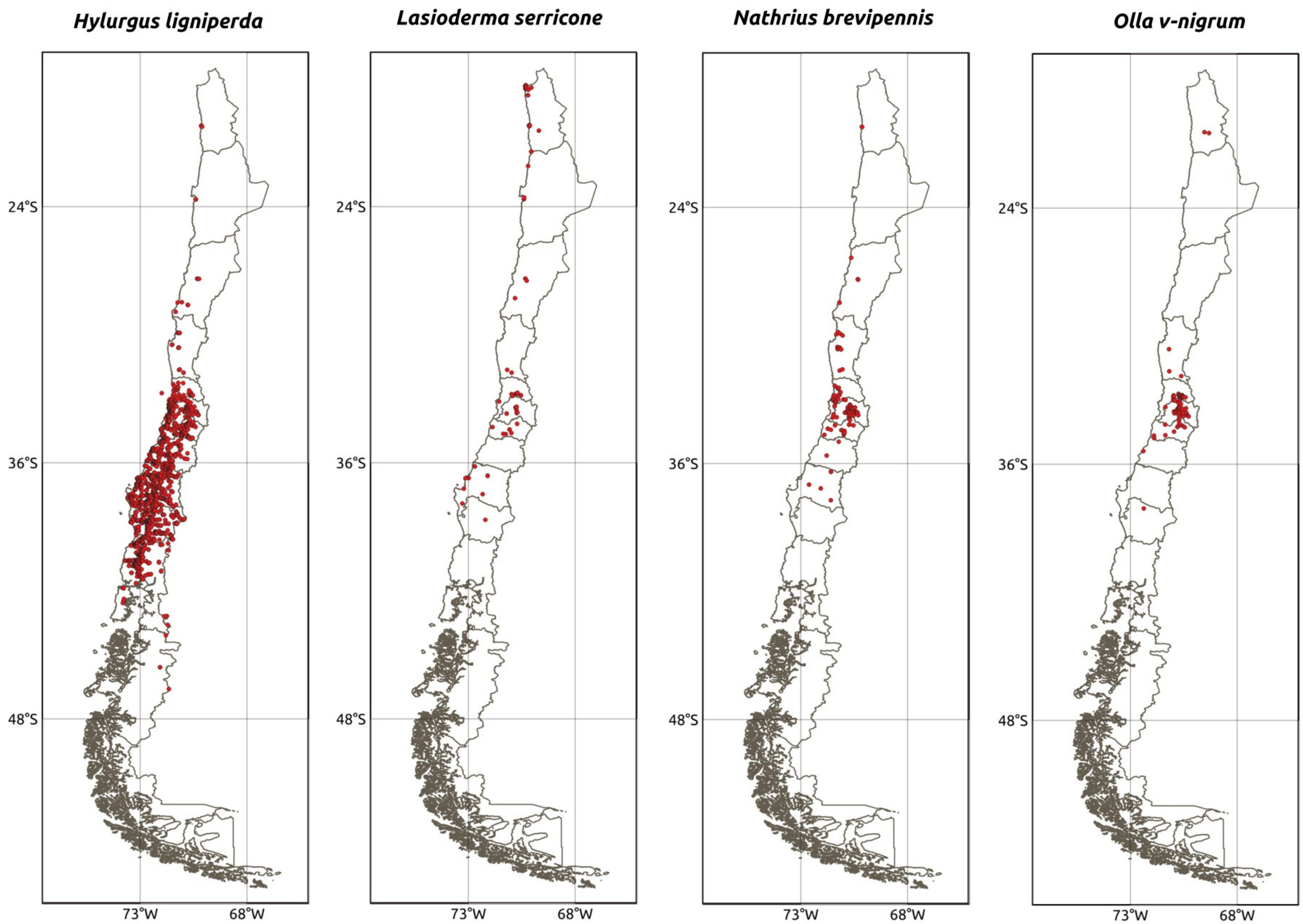

Hylurgus ligniperda
Lasioderma serricone
Nathrius brevipennis
Olla v-nigrum
24°S
36°S
48°S
73°W
68°W

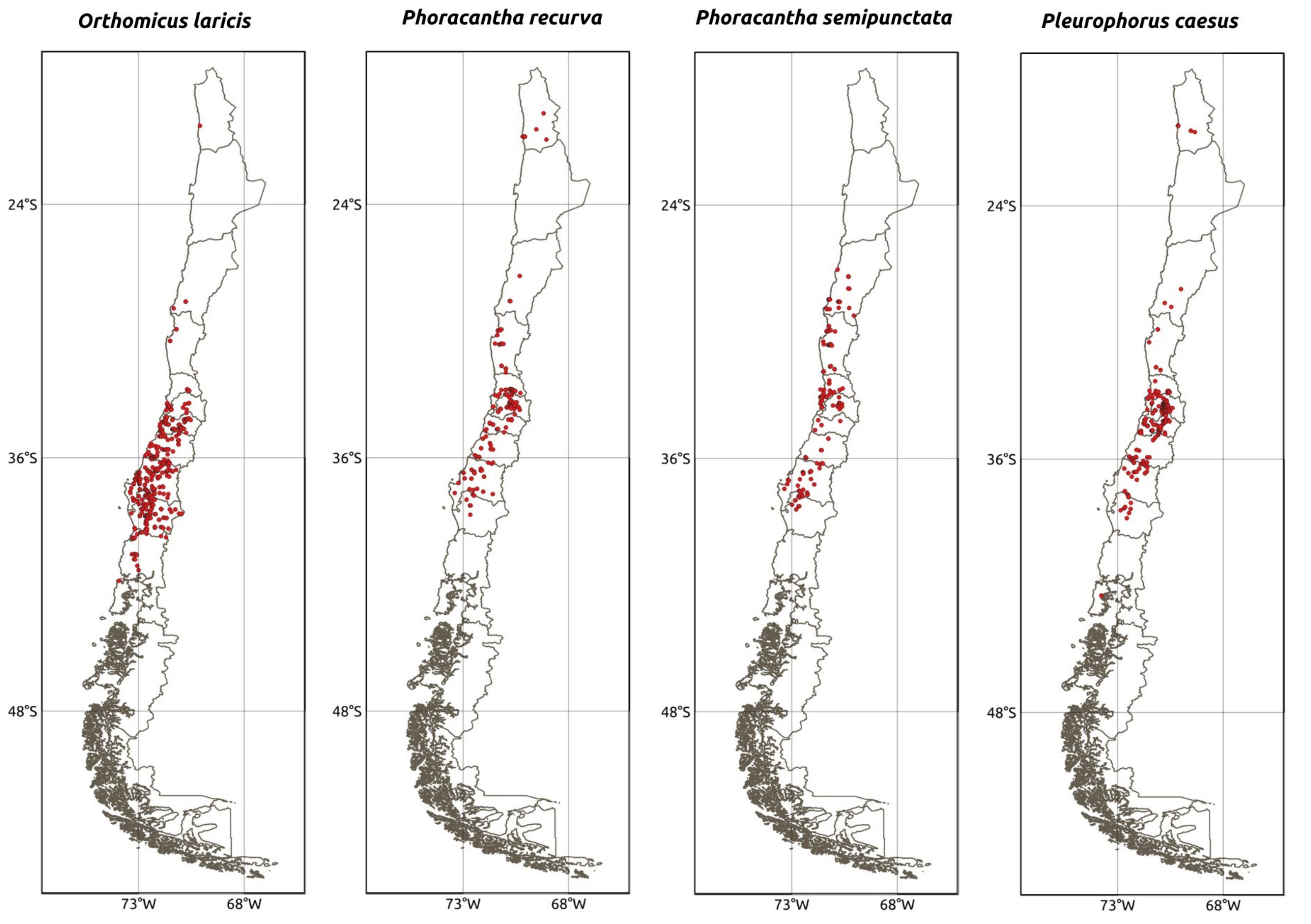

Orthomicus laricis
Phoracantha recurva
Phoracantha semipunctata
Pleurophorus caesus
24°S
36°S
48°S
73°W
68°W

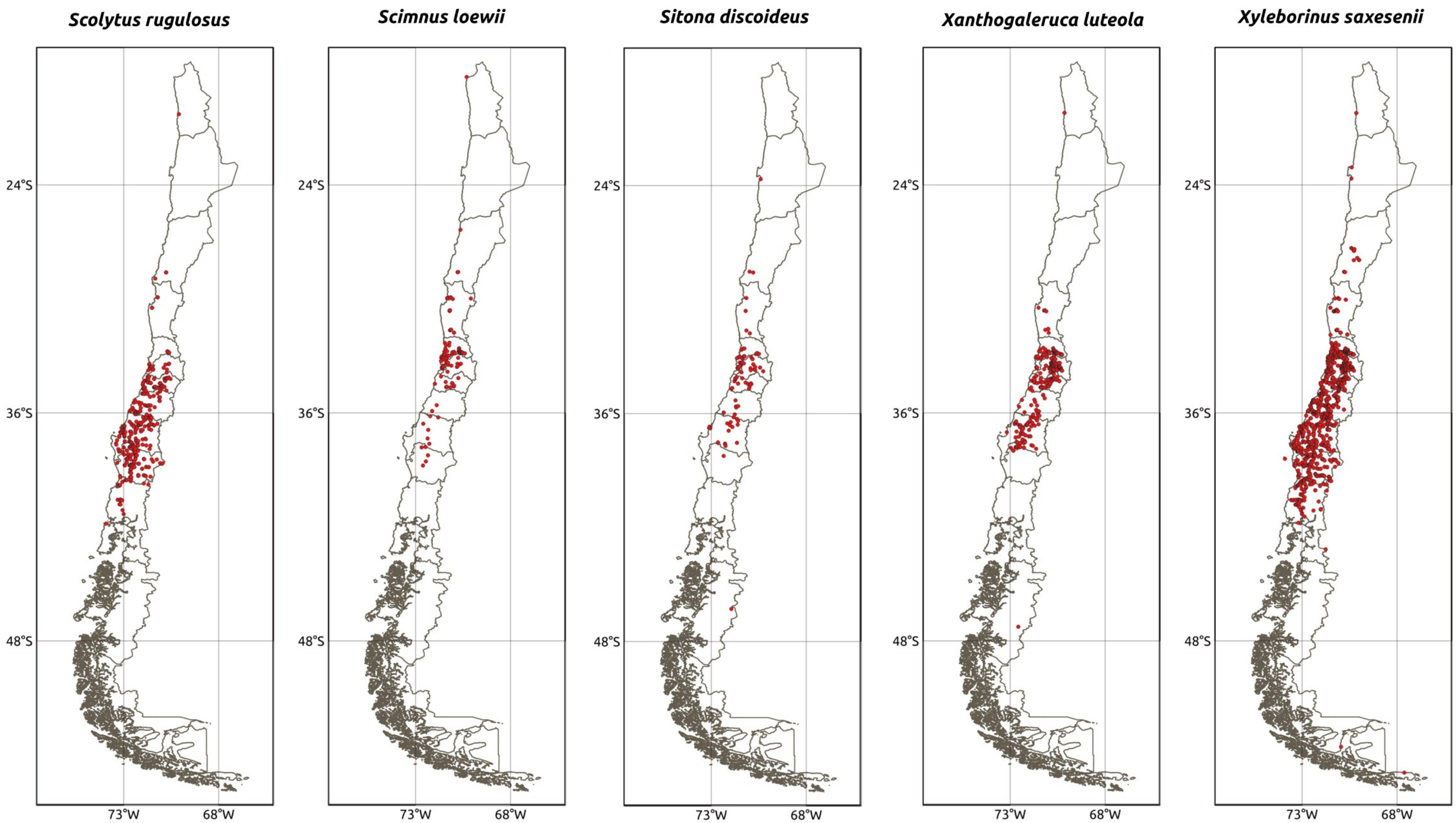
Scolytus rugulosus
Scimnus loewii
Sitona discoideus
Xanthogaleruca luteola
Xyleborinus saxesenii
24°S
36°S
48°S
73°W
68°W
